# Eosinophilic Cells in Ovarian Borderline Serous Tumors as a Predictor of BRAF Mutation

**DOI:** 10.3390/cancers16132322

**Published:** 2024-06-25

**Authors:** Alina Badlaeva, Anna Tregubova, Andrea Palicelli, Aleksandra Asaturova

**Affiliations:** 1 National Medical Research Center for Obstetrics, Gynecology and Perinatology Named after Academician V.I. Kulakov of the Ministry of Health of Russia, Bldg. 4, Oparina Street, 117513 Moscow, Russia; a_badlaeva@oparina4.ru (A.B.); a_tregubova@oparina4.ru (A.T.); 2 Pathology Unit, Azienda Unità Sanitaria Locale—IRCCS di Reggio Emilia, 42123 Reggio Emilia, Italy; andrea.palicelli@ausl.re.it

**Keywords:** eosinophilic cells, *BRAF* V600E mutation, ovarian serous borderline tumor

## Abstract

**Simple Summary:**

Ovarian serous borderline tumor (SBT) is a known precursor of low-grade serous carcinoma, and it has been reported that about 50% of SBTs are BRAF-mutated and that these tumors have a better prognosis. Therefore, early identification of the mutation is important for accurate treatment and follow-up of patients with SBT. It has been shown that eosinophilic cells (ECs) can be a histologic sign of a BRAF mutation. Therefore, the aim of our retrospective study was to evaluate the interobserver reproducibility for these cells. A BRAFV600E mutation was found in 45% of cases. The interobserver reproducibility in the assessment of ECs was substantial. The sensitivity and specificity for predicting the mutation were 79% and 91%, respectively, so ECs in ovarian SBTs can be used for initial screening of the BRAFV600E mutation to stratify patients and establish a prognosis.

**Abstract:**

According to recent reports, ovarian serous borderline tumor (SBT) harboring the *BRAF* V600E mutation is associated with a lower risk of progression to low-grade serous carcinoma. Preliminary observations suggest that there may be an association between eosinophilic cells (ECs) and the above-mentioned mutation, so this study aimed to evaluate interobserver reproducibility for assessing ECs. Forty-two samples of SBTs were analyzed for ECs with abundant eosinophilic cytoplasm. Immunohistochemical staining and genetic pro-filing were performed in all cases to verify the *BRAF* V600E mutation. A *BRAF* V600E mutation was found in 19 of 42 (45%) cases. Inter-observer reproducibility in the assessment of ECs was substantial (κ = 0.7). The sensitivity and specificity for predicting the mutation were 79% and 91%, respectively. Patients with *BRAF*-mutated SBTs were significantly younger than those without mutation (*p* = 0.005). SBTs with *BRAF* mutation were less likely to be accompanied by non-invasive implants than wild-type SBT: 12% (2/17) versus 33% (6/18). Seven cases were excluded due to incomplete cytoreductive surgery. Nevertheless, Fisher’s exact test showed no significant differences between the two groups (*p* = 0.228). Overall, this study strengthens the idea that ECs in ovarian SBTs may represent a mutation with prognostic significance, which can serve as a primary screening test for *BRAF* V600E mutation in this pathologic entity.

## 1. Introduction

Ovarian serous borderline tumor (SBT) can be defined as a low-grade, non-invasive neoplasm consisting of branched papillae lined by stratified serous epithelium [1]. The mean age of SBT is 50 years [1]. These tumors are usually unilateral and can exhibit intracystic growth or involve the ovarian surface.

Despite the excellent prognosis of FIGO stage I tumors, the likelihood of progression from SBT to low-grade serous carcinoma (LGSC) varies between 4 and 15% depending on the data [1,2,3,4,5]. In these cases, patients have a less-favorable prognosis, a poor clinical course, and poorer survival. To date, histological features such as a micropapillary growth pattern in SBTs indicate progression [1].

Recent studies confirm the important role of *KRAS* or *BRAF* mutations in SBTs, both of which are regulators of the mitogen-activated protein kinase (MAPK) signaling pathway. While the former can detect the development of low-grade serous carcinoma, the latter is associated with a lower risk of progression [5,6]. For example, a recent study by Chui et al. (2019) found that *KRAS*-mutated and wild-type SBTs have a risk of 2.3% and 4.4% risk of subsequent LGSC, respectively, whereas this risk is only 0.5% for *BRAF*-mutated SBTs [5].

In recent years, there has been increasing literature on specific morphologic features, including eosinophilic cells (ECs) with abundant eosinophilic cytoplasm, in *BRAF*-mutated SBTs [7]. These ECs can be defined as tumor cells with a senescence phenotype induced by the *BRAF* mutation. The mechanism for the formation of ECs is as follows: Activation of the *BRAF* oncogene leads to a significant reduction of the ribonucleotide reductase (RNR) subunit M2 (RRM2), resulting in cell cycle arrest via a decrease in deoxyribonucleotide triphosphate (dNTP) levels and alteration of DNA [8]. Subsequently, the activated BRAF oncogene can lead to oncogene-induced senescence (OIS), resulting in the formation of ECs [8,9]. This process is further confirmed by in vitro studies demonstrating the expression of senescence markers (such as senescence-associated beta-galactosidase and p16) and a low proliferation index (Ki-67) in ECs [10]. Taken together, these recent data suggest that *BRAF* mutation appears to play a crucial role in preventing progression from SBTs to LGSCs.

Studies have been performed on *BRAF*-mutated SBTs, but to the best of our knowledge, only one published article has investigated the reproducibility in identifying ECs in SBTs among pathologists [4]. Therefore, the aim of our study was to evaluate the interobserver reproducibility for ECs and the sensitivity and specificity rates for the assessment of this histologic feature.

## 2. Materials and Methods

Diagnostic slides were retrieved from 42 cases of ovarian SBTs from eligible female patients aged 15 to 79 years who had undergone cytoreductive surgery at the Research Center for Obstetrics, Gynecology, and Perinatology (Moscow, Russia) in calendar years 2019–2023. The histological slides had been cut from routinely processed, formalin-fixed, paraffin-embedded (FFPE) specimens. The exclusion criteria were insufficient or inappropriate samples (less than 50% tumor tissue; specimens with defects on microtome section or hematoxylin and eosin staining). No micropapillary SBTs were included in our series. In seven cases, incomplete cytoreductive surgery was performed because no frozen section diagnosis was available.

Histologic slides were independently assessed by 3 gynecologic pathologists to confirm the diagnosis and evaluate the presence of *BRAF* mutation-associated histologic features: round cells with abundant dense/glassy eosinophilic cytoplasm occupying at least 50% of the cell area. These ECs were freely detected at 100× magnification and were located within the epithelium or detached from the surface of the epithelium. ECs were binary scored as present or absent, blinded to *BRAF* mutation status. All participating pathologists were trained to recognize ECs using example images taken from publications describing basic ECs research conducted at Johns Hopkins University School of Medicine and Memorial Sloan Kettering Cancer Center [4,5].

All cases were analyzed for a *BRAF* V600E mutation via immunohistochemistry (IHC) and molecular analysis. IHC staining for the mutation was performed with the VENTANA OptiView DAB IHC Detection Kit (Ventana Medical-Systems, Oro Valley, AZ, USA) on the BenchMark XT automated immunostainer (Ventana Medical-Systems, Oro Valley, AZ, USA) using a polyclonal mouse anti-BRAF antibody (dilution 1:50, ABclonal, Wuhan, China). IHC was classified as positive if cytoplasmic staining with the antibody was present in ≥50% of neoplastic cells.

A *BRAF* V600E mutation was detected, caused by the transversion T→A at nucleotide 1799 (T1799A) in codon 600, where an amino acid substitution of valine (V) to glutamic acid (E) in the activation segment of the kinase domain generates a constitutively active form of the protein. Genotyping was performed via Sanger sequencing using the Genetic Analyzer 3500 (Applied Biosystems, Foster City, CA, USA). DNA extraction was performed using the QIAamp DNA FFPE Tissue Kit (Qiagen, Santa Clarita, CA, USA). Table 1 shows the primers used to identify mutations in exon 15 of the *BRAF* gene via polymerase chain reaction (PCR).

**Figure 1:**
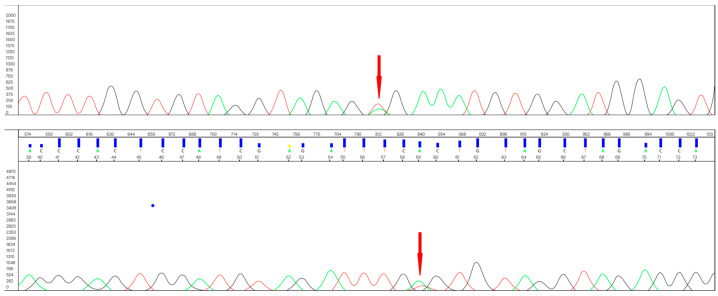
The sequence of *BRAF* mutation (the colors stand for the DNA. nitrogenous bases: A-green, G-black, T-red, C-blue).

The nucleotide sequences obtained (Figure 1) were analyzed using Sequencing Analysis 6 software (Applied Biosystems).

Comparisons between the two groups (*BRAF*-mutated and wild-type) were performed using Mann–Whitney and Fisher’s exact tests. Fleiss’ kappa and Cohen’s kappa were calculated to perform an analysis of inter-rater reliability analysis and to measure interobserver agreement in the assessment of ECs. The consensus score for validation of reproducibility (presence of ECs/absence of ECs) was determined by 2 of 3 pathologists. F1 score was calculated, defined as the harmonic mean of precision and recall: 2 × (Precision × Recall)/(Precision + Recall), where Precision = True Positive/(True Positive + False Positive); Recall = True Positive/(True Positive + False Negative). Sensitivity, specificity, and positive and negative predictive value were estimated to measure the accuracy of the ECs. A *p*-value < 0.05 was considered significant.

## 3. Results

According to the genetic testing, approximately one half of the SBTs harbored *BRAF* mutation (19 of 42 cases, 45%). This mutation consisted in all cases of the usual T to A substitution at position c.1799 (c.1799T > A) in exon 15 of the coding DNA.

The clinical features of the SBTs, stratified by genotype, are summarized in Table 2.

The data in Table 2 show that the patients in the *BRAF*-mutated group were significantly younger at the time of surgery than those in the wild-type SBT group (*p* < 0.05). The median age of patients in the first group was 33.6 years, while the median age of patients with wild-type SBTs was 43.9 years.

SBTs with mutation were less likely to have non-invasive implants in the omentum and peritoneum than wild-type patients: 12% and 33%, respectively, although this difference was not statistically significant. Of 35 SBTs, 2 mutated and 6 wild-type neoplasms were at an advanced FIGO stage (higher than I). In seven cases, information about the FIGO stage was not accessible because no omentum resection and no peritoneal biopsy and smears were performed. Incomplete cytoreductive surgery was performed because the ovarian tumors were suspected to be benign, and no frozen section diagnosis was made.

Mid-term follow-up of 5 years was available for 19 patients, 2 of whom had tumor recurrence (one case in each group). Of 14 cases with short-term follow-up (3 years), there was 1 recurrence in the wild-type group.

ECs were identified by the majority of pathologists (2 or 3) in 79% of *BRAF*-mutated (15/19) and in 9% (2/23) wild-type tumors (*p* < 0.0001). The interobserver reproducibility of the presence of ECs was substantial (κ = 0.7, 95% confidence interval, CI 0.692–0.703). The overall F1 score was 0.83. The results of Cohen’s Kappa are shown in Table 3.

Table 4 illustrates the interobserver variation of EC scores assigned by 3 pathologists and the percent of agreement for all the cases, which were sorted via *BRAF* mutation status.

**Figure 2:**
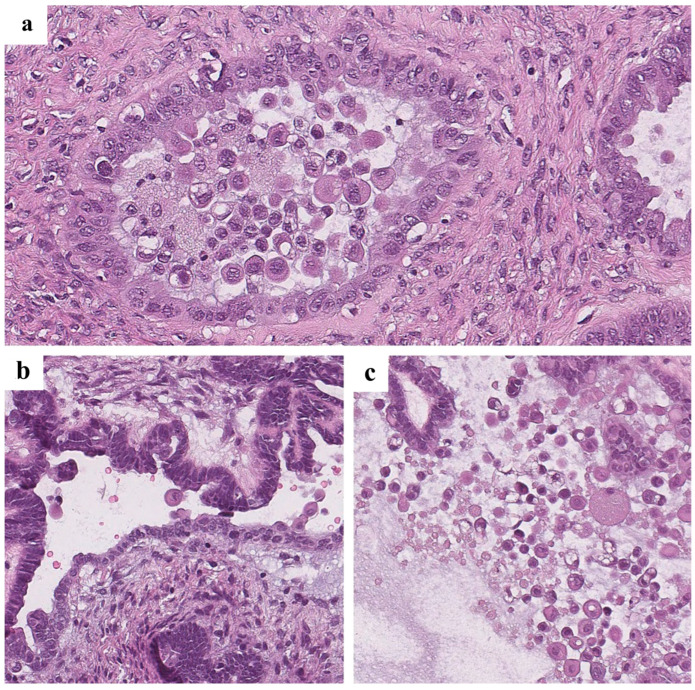
Eosinophilic cells in *BRAF*-mutated cases with excellent agreement between reviewers (previously unpublished, original photos). Hematoxylin and eosin staining ((**a**) magnification 200×, (**b**,**c**) magnification 100×). There were two cases (10%) of mutated neoplasms in which all three reviewers found no ECs. At the same time, there were two cases in the wild-type group in which all reviewers found ECs (Figure 3).

The data in Table 4 show that in 53% (10/19) of *BRAF*-mutated tumors, the agreement between all reviewers was perfect. Notable examples of these cases are presented in Figure 2.

**Figure 4:**
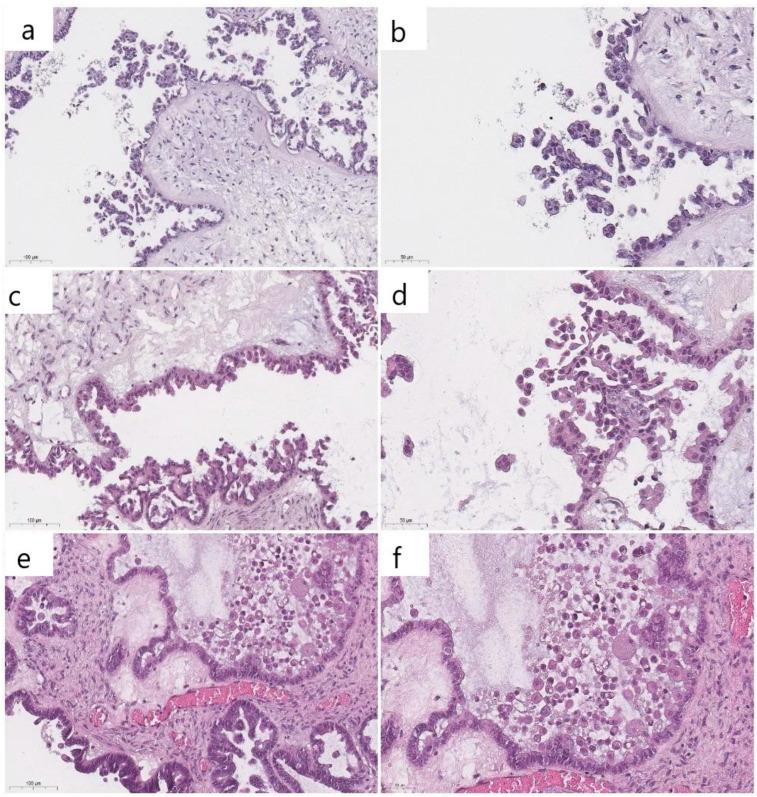
Overlapping morphology of epithelial cells detaching from the tumor surface and resembling eosinophilic cells (**a**–**d**) and classic eosinophilic cells (**e**,**f**) (previously unpublished, original photos). Hematoxylin and eosin staining (Magnification 100×).

In several cases of wild-type SBTs, there were stratified and tufted cells that were mistaken for bona-fide ECs; as a result, there was substantial reproducibility among pathologists. Both cell types were frequently desquamated from the tumor surface and floated freely above the epithelial layer. However, when comparing the histologic features of classic ECs and their mimics, the latter were found to be smaller and lack abundant eosinophilic cytoplasm (Figure 4).

For ECs, the sensitivity and specificity for predicting a *BRAF* V600E mutation were 78.9% (95% CI 56.7–91.5) and 91.3% (95% CI 73.2–98.5), respectively. The positive predictive value was 88.2% (95% CI 65.7–97.9); the negative predictive value 84% (95% CI 73.2–98.5).

**Figure 5:**
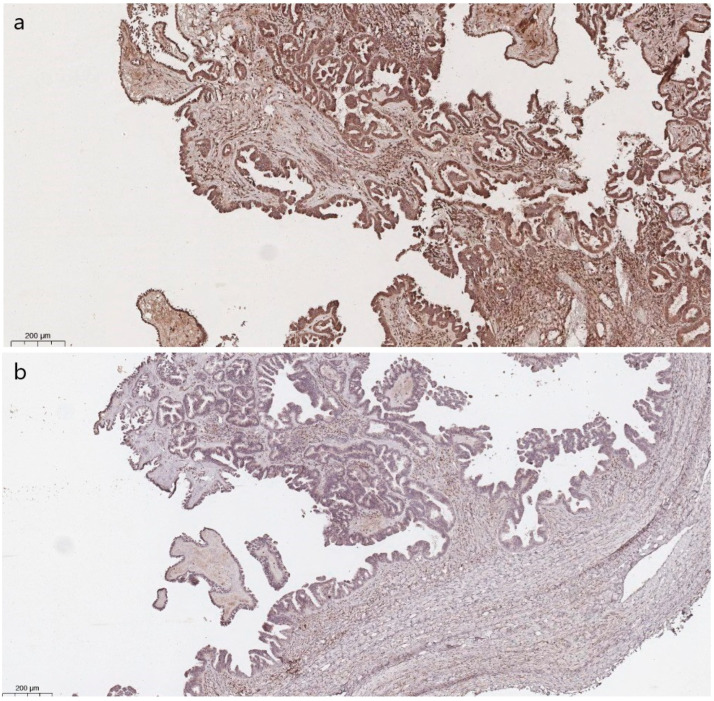
Positive cytoplasmic staining in ≥50% neoplastic cells in *BRAF*-mutated SBT (**a**) and negative expression in wild-type SBT (**b**) (previously unpublished, original photos). Immunohistochemical staining with polyclonal anti-BRAF antibody.

There was no significant difference in immunohistochemical staining between the groups (*p* = 0.15). Nevertheless, wild-type SBTs showed cytoplasmic staining less frequently than *BRAF*-mutated ones (Figure 5).

While the former showed a positive reaction with the BRAF antibody in 26% of cases, the latter showed a positive reaction in 47% of cases. The sensitivity and specificity of the IHC were 47% (95% CI 27.3–68.3) and 74% (95% CI 53.5–87.4), respectively.

## 4. Discussion

SBT is known as an indolent ovarian neoplasm, leading to long-term follow-up to distinguish clinical outcomes [1,3,11]. This fact makes the prognosis for SBTs a major challenge. A number of studies have suggested that the most critical endpoint is progression to LGSC and have found that *BRAF* mutation status is related to the favorable clinical behavior of SBTs [3,5,6].

Several observations have also shown that not only the prevention of lesion progression but also oncogene-induced senescence is related to *BRAF* mutation status [7,8,9,10]. Oncogene-induced senescence has been found to lead to cell growth arrest in the G1 phase of the cell cycle, the stage at which most normal cells undergo replicative senescence [12]. The slowing of G1 growth was confirmed by specific changes in cyclins, i.e., a decrease in the expression of cyclin B1 and an increase in the expression of cyclins D1 and D2 [12].

A phenomenon of spontaneous cell senescence has been demonstrated in ovarian cancer cells exposed to poly (ADP-ribose) polymerase inhibitors such as olaparib [13]. As with cell cycle inhibitors, senescent epithelial ovarian tumors also show increased expression of p16, p21, and p53 [4,7,10,14].

It is noteworthy that the senescence-induced immunophenotype is typical of many other neoplasms. This was observed in Reed–Sternberg cells from Hodgkin’s lymphoma, which exhibited features of senescence [15]. High levels of p16 were also observed in spontaneous senescence of precancerous lesions in the lungs of K-RAS V12 mice [16].

This study has demonstrated that *BRAF*-mutated SBTs are strongly associated with senescent cells with abundant dense eosinophilic cytoplasm. This finding is consistent with the results of previous studies [4,5,7,10]. The data obtained in the present work showed that histomorphological assessment of ECs had significant sensitivity and specificity (78.9% and 91.3%, respectively) for the prediction of *BRAF* V600E mutation in all reviewers. This is supported by the study of Chui (2023), which showed similar results: the median sensitivity and specificity for identifying *BRAF*-mutated SBTs were 67% and 95%, respectively [4].

The results of the agreement between the three reviewers showed that Fleiss’ kappa was 0.7. This result indicates considerable reproducibility in the estimation of ECs between observers. These results differ from Chui’s estimate of moderate inter-rater agreement between five observers, which found Fleiss’ kappa to be 0.41 [4]. The above differences can be partly explained by the fact that more participating researchers were included and a semi-quantitative assessment of the extent of ECs was used, whereas a binary assessment was used in this study. In addition, a possible influence of the histological subtype of the tumor cannot be excluded. The lower interobserver reproducibility might be related to a morphologic mimic such as micropapillary SBT; this growth pattern was absent in the present study, whereas in Chui’s study, 12 samples showed micropapillary features [4].

Discordant results in interobserver reproducibility could also be attributed to cuboidal and columnar cells lining the papillae of SBTs, which could be mistaken for ECs [4,7,10]. It could, therefore, be the case that these tufting or hobnail tumor cells can mimic ECs. However, the latter are rounder, with more abundant eosinophilic cytoplasm, lacking cilia and mitotic figures [10]. To address this issue, several studies had previously indicated that IHC could be a powerful, specific, and sensitive tool with which to identify *BRAF* V600E mutation in serous ovarian tumors [4,7,17]. Contrary to expectations, this study found no significant difference in BRAF staining between the two groups and demonstrated the low sensitivity and specificity of the IHC method. However, these results must be interpreted with caution as this rather contradictory result could be due to the use of a polyclonal anti-BRAF antibody, while in other studies, the VE1 clone was used [4,7,17]. Therefore, it can be assumed that the detection of true ECs might be a challenge for pathologists.

The most important role of *BRAF* mutation status and, consequently, ECs is as a prognostic value for the follow-up of SBT patients. The stepwise pathogenesis of LGSCs was first described by R. Kurman et al., who detected the same *BRAF* and *KRAS* mutations in the regions of serous cystadenomas adjacent to SBTs [18]. It was then shown that LGSCs can also develop from the expansion of *KRAS*-mutated clones in SBTs [11,19,20]. Moreover, *KRAS* mutations are frequently detected in recurrent LGSCs [21]. Furthermore, that certain molecular features are associated with a high or low risk of progression from SBTs to LGSCs has been investigated [5,22,23,24].

Consequently, surrogate markers that may reflect the activity of key driver genes in the progression from SBTs to LGSCs have been tested as a prognostic factor to improve the personalized approach in the follow-up period for patients with SBTs, especially in those of reproductive age [6,25,26,27,28,29]. The components of the MAPK-related signaling pathway (KRAS, BRAF, and NRAS) have been used differently in routine practice: while the *KRAS* immunohistochemical marker showed both low sensitivity (27%) and low specificity (64%) in detecting the *KRAS* mutation, *BRAF* immunostaining showed perfect sensitivity and specificity in detecting the V600E mutation when a monoclonal antibody (VE1 clone) was used [25,26,27]. Since these results were obtained, *BRAF* mutation status has been successfully tested as a prognostic factor in several studies [5,28,29]. *KRAS* testing with genotyping has also demonstrated prognostic value for SBTs as it is a reliable tool for recurrence prediction [6].

In addition, Hunter et al. [30] used genome-wide, high-resolution copy number analysis to identify driver genes and predict the clinical behavior of SBTs and LGSCs. Although the overall copy number aberrations (CNA) were demonstrated to be higher in LGSCs, SBTs also had equal aberration levels to LGSCs. Among these aberrations, loss of chromosome 9p and homozygous deletions of the *CDKN2A* locus were considered significant. Homozygous deletion of chromosome 9p21, which includes the CDKN2A gene, usually leads to co-deletion of adjacent genes, resulting in inactivation of methylthioadenosine phosphorylase (MTAP) in the vast majority of tumors [31,32,33]. Immunohistochemical assessment of MTAP as a prognostic marker for SBTs showed a correlation between loss of MTAP expression in SBTs and unfavorable clinical behavior and potential progression to LGSCs; about 2/3 of bilateral SBTs, i.e., all SBTs with micropapillary patterns and the SBTs with lymph node involvement, showed MTAP to be a reliable immunohistochemical marker for SBTs prognosis [34].

In addition to the components of the MAPK-signaling pathway and other molecular subtype-related markers, several other widely used immunohistochemical antibodies were also tested, which should be introduced into routine pathology practice due to their high availability and cost-effectiveness. Initially, the Ki-67 labeling index was investigated to predict benign follow-up for SBTs patients and to verify SBTs with a high risk of progression to LGSCs. Although many authors have demonstrated a stepwise increase in proliferative activity from benign serous cystadenomas to SBTs and, further, to LGSCs, they failed to identify a reliable cut-off value for Ki-67 with high sensitivity and specificity for the prediction of progression from SBTs to LGSCs [35,36,37].

Steroid receptors have also been proposed as a beneficial prognostic marker [38,39,40,41], but recent literature has provided conflicting results. While some studies found that steroid receptor status can be used as a prognostic factor [42], the others could not confirm any prognostic value [43], or only one of the steroid receptors (progesterone or estrogen) showed positive prognostic potential [44,45]. Nevertheless, the cut-off value and method for assessing steroid receptor status have yet to be finalized and are still not recommended to be included in the guidelines and protocols for the diagnosis and treatment of SBTs.

Among many other immunohistochemical markers tested (p53, p16, ER, PR, PTEN, PAX2, Mammaglobin, RB1, Cyclin E1, stathmin, LMP2, L1CAM, CD44, PAX8, ARID1A, HNF1B, Napsin A, CDX2, SATB2, MUC4, BRG1, AMACR, TTF1, BCOR, NTRK), no significant differences were found between LGSCs and SBTs [44].

Based on the above, several studies have produced estimates of different potential prognostic biomarkers [35,36,37,38,39,40,41,42,43,44,45], but there is still insufficient data to determine the most valid one.

## 5. Conclusions

ECs are reliable markers for *BRAF* mutation status that do not require any additional methods for testing. This histological feature has enormous potential as an effective prognostic marker for the progression of SBTs compared to other immunohistochemical markers, as their expression is not unique and the scale for scoring is not standardized. Thus, the correct identification of ECs could drastically contribute to prognosis and a clinical treatment strategy, especially in advanced-stage patients.

## Figures and Tables

**Figure 3:**
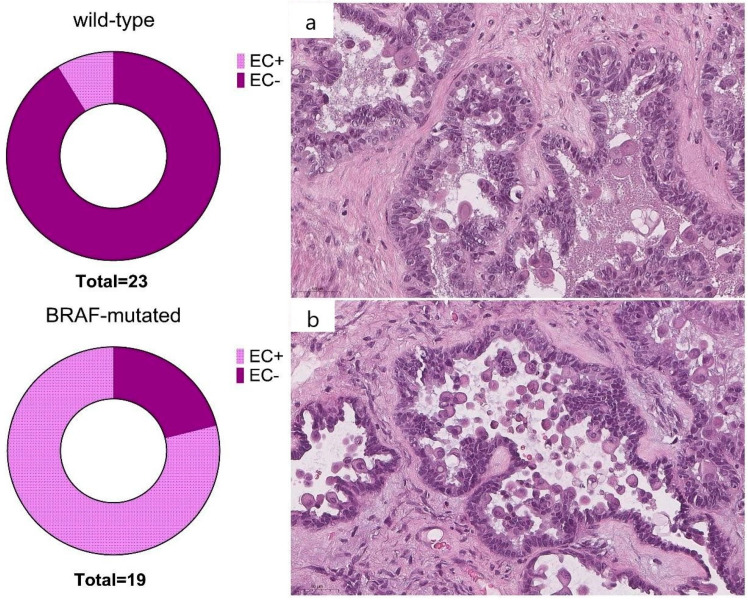
A population of cells with discrete borders, abundant eosinophilic cytoplasm, and round nuclei (eosinophilic cells) in both wild-type (**a**) and *BRAF*-mutation SBTs (**b**) (previously unpublished, original photos). Hematoxylin and eosin staining (Magnification 100×).

**Table 1:** Used primers to analyze exon 15 of *BRAF* gene.

Primers	Primer Sequence	Annealing Temperature (°C)
Forward primer (F)	5′-AATGCTTGCTCTGATAGGAA	58
Reverse primer (R)	5′-TCAGTGGAAAATAGCCTCAAT	58

**Table 2:** Clinical features of serous borderline tumors stratified by genotype.

Feature		Wild-Type	(n)	Mutant-Type	(n)	Total (n)	*p*-Value
Age (median, Q1–Q3)		43.9 (34–57)	23	33.6 (23–38)	19	42	0.049
FIGO stage I	IA	7 (58.3%)	12	15 (100%)	15	27	0.128
	IB	5 (41.7%)		0			
	IC	0		0			
FIGO stage > I	IIA	1 (16.7%)	6	1 (50%)	2	8	
	IIB	2 (33.3%)		0			
	IIIA	0		0			
	IIIB	3 (50%)		1 (50%)			
	IIIC	0		0			
	IV	0		0			
Short-term follow-up		1 (14.3%)	7	0	7	14	0.299
Mid-term follow-up		1 (9%)	11	1 (12.5%)	9	19	0.811

**Table 3:** Pairwise agreement values.

	Pathologist 1	Pathologist 2	Pathologist 3
**Pathologist 1**		0.793	0.652
**Pathologist 2**			0.652
**Results of genetic test**	0.608	0.608	0.759

**Table 4:** Eosinophilic cell scores in *BRAF*-mutated and wild-type serous borderline tumors determined by each pathologist.

Case No.	*BRAF* Mutation	Pathologist 1	Pathologist 2	Pathologist 3	% of Agreement
1	Wild-type	0	0	0	100
2	Wild-type	0	0	0	100
3	p.V600E (c.1799T > A)	1	1	1	100
4	p.V600E (c.1799T > A)	0	0	0	100
5	p.V600E (c.1799T > A)	1	1	1	100
6	p.V600E (c.1799T > A)	0	1	1	66.7
7	Wild-type	0	0	0	100
8	p.V600E (c.1799T > A)	1	1	1	100
9	Wild-type	0	0	0	100
10	Wild-type	0	0	0	100
11	p.V600E (c.1799T > A)	1	1	1	100
12	Wild-type	0	0	0	100
13	Wild-type	1	1	0	66.7
14	Wild-type	0	0	0	100
15	Wild-type	0	0	0	100
16	p.V600E (c.1799T > A)	0	1	1	100
17	Wild-type	0	0	0	100
18	Wild-type	0	0	0	100
19	p.V600E (c.1799T > A)	1	1	1	100
20	Wild-type	0	0	0	100
21	p.V600E (c.1799T > A)	0	0	0	100
22	p.V600E (c.1799T > A)	1	1	1	100
23	p.V600E (c.1799T > A)	1	1	1	100
24	p.V600E (c.1799T > A)	1	1	1	100
25	Wild-type	0	0	0	100
26	Wild-type	0	0	1	33.3
27	Wild-type	0	0	0	100
28	Wild-type	0	0	0	100
29	Wild-type	0	0	0	100
30	p.V600E (c.1799T > A)	1	1	0	66.7
31	p.V600E (c.1799T > A)	0	0	1	33.3
32	p.V600E (c.1799T > A)	1	1	1	100
33	Wild-type	1	1	1	100
34	p.V600E (c.1799T > A)	1	0	1	66.7
35	Wild-type	0	0	0	100
36	Wild-type	0	0	0	100
37	p.V600E (c.1799T > A)	1	0	1	66.7
38	Wild-type	0	0	0	100
39	p.V600E (c.1799T > A)	0	0	1	33.3
40	p.V600E (c.1799T > A)	1	1	1	100
41	Wild-type	0	0	0	100
42	Wild-type	0	0	0	100

## Data Availability

Data supporting reported results can be presented upon reasonable request.
